# The nasal mycobiome of individuals with allergic rhinitis and asthma differs from that of healthy controls in composition, structure and function

**DOI:** 10.3389/fmicb.2024.1464257

**Published:** 2024-12-17

**Authors:** Marcos Pérez-Losada, Eduardo Castro-Nallar, Jenaro García-Huidobro, José Laerte Boechat, Luis Delgado, Tiago Azenha Rama, Manuela Oliveira

**Affiliations:** ^1^Department of Biostatistics and Bioinformatics, Computational Biology Institute, Milken Institute School of Public Health, The George Washington University, Washington, DC, United States; ^2^CIBIO-InBIO, Centro de Investigação em Biodiversidade e Recursos Genéticos, Universidade do Porto, Campus Agrário de Vairão, Vairão, Portugal; ^3^Departamento de Microbiología, Facultad de Ciencias de la Salud, Universidad de Talca, Talca, Chile; ^4^Centro de Ecología Integrativa, Universidad de Talca, Talca, Chile; ^5^Centro de Investigaciones Médicas, Escuela de Medicina, Universidad de Talca, Talca, Chile; ^6^Serviço de Imunologia Básica e Clínica, Departamento de Patologia, Faculdade de Medicina da Universidade do Porto, Porto, Portugal; ^7^Centro de Investigação em Tecnologias e Serviços de Saúde (CINTESIS@RISE), Faculdade de Medicina da Universidade do Porto, Porto, Portugal; ^8^Serviço de Imunoalergologia, Unidade Local de Saúde São João (ULS São João), Porto, Portugal; ^9^UCIBIO, Research Unit on Applied Molecular Biosciences, Forensic Sciences Research Laboratory, University Institute of Health Sciences (1H-TOXRUN, IUCS-CESPU), Avenida Central de Gandra, Gandra, Portugal; ^10^Associate Laboratory i4HB—Institute for Health and Bioeconomy, University Institute of Health Sciences—CESPU, Avenida Central de Gandra, Gandra, Portugal

**Keywords:** allergy, asthma, ITS, mycobiome, nasal cavity, Portugal, rhinitis

## Abstract

Allergic rhinitis (AR) and asthma (AS) are two of the most common chronic respiratory diseases and a major public health concern. Multiple studies have demonstrated the role of the nasal bacteriome in AR and AS, but little is known about the airway mycobiome and its potential association to airway inflammatory diseases. Here we used the internal transcriber spacers (ITS) 1 and 2 and high-throughput sequencing to characterize the nasal mycobiome of 339 individuals with AR, AR with asthma (ARAS), AS and healthy controls (CT). Seven to ten of the 14 most abundant fungal genera (*Malassezia, Alternaria, Cladosporium, Penicillium, Wallemia, Rhodotorula, Sporobolomyces, Naganishia, Vishniacozyma*, *a*nd *Filobasidium*) in the nasal cavity differed significantly (*p* ≤ 0.049) between AS, AR or ARAS, and CT. However, none of the same genera varied significantly between the three respiratory disease groups. The nasal mycobiomes of AR and ARAS patients showed the highest intra-group diversity, while CT showed the lowest. Alpha-diversity indices of microbial richness and evenness only varied significantly (*p* ≤ 0.024) between AR or ARAS and CT, while all disease groups showed significant differences (*p* ≤ 0.0004) in microbial structure (i.e., beta-diversity indices) when compared to CT samples. Thirty metabolic pathways (PICRUSt2) were differentially abundant (Wald’s test) between AR or ARAS and CT patients, but only three of them associated with 5-aminoimidazole ribonucleotide (AIR) biosynthesis were over abundant (log2 Fold Change >0.75) in the ARAS group. AIR has been associated to fungal pathogenesis in plants. Spiec-Easi fungal networks varied among groups, but AR and ARAS showed more similar interactions among their members than with those in the CT mycobiome; this suggests chronic respiratory allergic diseases may disrupt fungal connectivity in the nasal cavity. This study contributes valuable fungal data and results to understand the relationships between the nasal mycobiome and allergy-related conditions. It demonstrates for the first time that the nasal mycobiota varies during health and allergic rhinitis (with and without comorbid asthma) and reveals specific taxa, metabolic pathways and fungal interactions that may relate to chronic airway disease.

## 1 Introduction

Allergic rhinitis and asthma are two of the most common chronic airway diseases in Western countries inflicting a relevant health and economic burden to society (Todo-Bom et al., 2007; Sa-Sousa et al., 2012; Fonseca et al., 2021). In Portugal, allergic rhinitis has a prevalence of 9–10% in children and adolescents and 26.1% in adults (Todo-Bom et al., 2007; Falcão et al., 2008; Muc et al., 2014); while asthma has a prevalence of 8.4% in children and adolescents and 6.8% in adults (Sa-Sousa et al., 2012; Muc et al., 2014; Ferreira-Magalhaes et al., 2016).

Allergic rhinitis is considered an inflammation of the nasal mucosa, characterized by sneezing, congestion, itching, and rhinorrhea (Steelant et al., 2016; Steelant et al., 2018; Acevedo-Prado et al., 2022; Savoure et al., 2022). Similarly, asthma is a multifactorial condition of the airways characterized by obstruction, inflammation, and mucous production (Mims, 2015; Licari et al., 2018; Dharmage et al., 2019). Allergic rhinitis and asthma frequently coexist (Compalati et al., 2010; Pite et al., 2014; Ferreira-Magalhaes et al., 2015; Small et al., 2018; Bousquet et al., 2019)—more than 46% of the Portuguese patients with asthma also show allergic rhinitis (Valero et al., 2009; Acevedo-Prado et al., 2022). This suggests that they may represent a combined airway inflammatory disease with several pathophysiological, epidemiological, and clinical connections within the concept of a united airway disease (Bergeron and Hamid, 2005; Pawankar, 2006; Kim et al., 2008; Compalati et al., 2010; Bousquet et al., 2023).

Multiple metataxonomic and metagenomic studies have already demonstrated that the upper airway bacteriome is a gatekeeper of respiratory health and plays a significant role in the onset, development, and severity of both allergic rhinitis (Lal et al., 2017; Bender et al., 2020; Gan et al., 2021; Chen et al., 2022; Kim et al., 2022; Azevedo et al., 2023; Pérez-Losada et al., 2023a,b) and asthma (Bogaert et al., 2011; Brar et al., 2012; Huang and Boushey, 2014; Castro-Nallar et al., 2015; Dickson and Huffnagle, 2015; Huang and Boushey, 2015; Pérez-Losada et al., 2015; Teo et al., 2015; Pérez-Losada et al., 2016a,b; Pérez-Losada et al., 2017; Dinwiddie et al., 2018; Frati et al., 2018; Pérez-Losada et al., 2018; Hufnagl et al., 2020; Losol et al., 2021; Raita et al., 2021). These same studies have also shown that the nasal cavity is a major reservoir for opportunistic bacterial pathogens, which can spread to other sections of the respiratory tract and potentially induce respiratory illnesses (Garcia-Rodriguez and Fresnadillo Martinez, 2002; Hilty et al., 2010; Bogaert et al., 2011; Dickson et al., 2013; Biesbroek et al., 2014; Huang and Boushey, 2015; Pérez-Losada et al., 2015; Teo et al., 2015; Pérez-Losada et al., 2016b; Prevaes et al., 2016; Huang, 2017; Lal et al., 2017; Pérez-Losada et al., 2017; Esposito and Principi, 2018; Pérez-Losada et al., 2018; Gan et al., 2021; Chen et al., 2022; Kim et al., 2022).

Less is known, however, about the human mycobiome and its role in chronic airway diseases (Goldman et al., 2018; Rick et al., 2020; van Tilburg Bernardes et al., 2020; Oliveira et al., 2023). The recent inclusion of fungi in human microbiome research has revealed that they are also implicated in asthma onset and development in susceptible individuals (Carpagnano et al., 2016; Goldman et al., 2018; Rick et al., 2020; van Tilburg Bernardes et al., 2020; Yuan et al., 2023), although very few studies have surveyed the upper airways (Jung et al., 2015; Yuan et al., 2023). Similarly, to the best of our knowledge, only one study so far has characterized the airway mycobiome of patients with allergic rhinitis (Jung et al., 2015); hence, the taxonomic composition and interactions, and functional diversity of the fungal communities inhabiting the nose remain unknown, or poorly understood at best, in both asthmatic and rhinitic patients.

In this study, we have used the internal transcriber spacer (ITS) 1 and 2 and next-generation sequencing to characterize the nasal mycobiomes of children and adults with allergic rhinitis (with and without asthma comorbidity), asthma and healthy controls. We describe unique fungal taxonomic and functional profiles across those four clinical groups and compare their composition, structure, metabolism, and network interactions.

## 2 Materials and methods

### 2.1 Participants

All participants enrolled in this study were part of the ASMAPORT Project (PTDC/SAU-INF/27953/2017). This study was approved by the “Comissão de Ética para a Saúde” of the Centro Hospitalar Universitário São João/Faculdade de Medicina (Porto, Portugal) in March 2017, Parecer_58-17. Written consent was obtained from all independent participants or their legal guardians using the informed consent documents approved by the Comissão de Ética.

ASMAPORT was a cross-sectional study of Portuguese children and adults designed to investigate host-microbe during asthma and rhinitis. Participants were recruited from northern Portugal while attending the outpatient clinic of the Serviço de Imunoalergologia in the Centro Hospitalar Universitário São João from July 2018 to January 2020. Healthy volunteers from the Porto area with no history of respiratory illness were also enrolled but did not complete the questionnaire or provide clinical information. The diagnosis of allergic rhinitis was confirmed by an allergy specialist based on clinical criteria and a positive skin prick or specific IgE test to at least one clinically relevant inhalant allergen in the region (Pereira et al., 2006; Bousquet et al., 2009). Diagnosis of asthma was confirmed by the attending physician based in the presence of typical symptoms in the previous 12 months or a positive bronchodilator responsiveness testing with salbutamol (Silva et al., 2019). Further details are provided in Pérez-Losada et al. (2023a,b).

### 2.2 Sampling

A total of 339 individuals participated in this study (Supplementary Table S1). They were categorized into four clinical groups: allergic rhinitis (AR = 47), allergic rhinitis with asthma (ARAS = 155), asthma (AS = 12), and healthy controls (CT = 125 individuals). Samples were collected by swabbing the right and left nostrils. Further detail is provided in Pérez-Losada et al. (2023a). Because of the sample size of the AS group, we have only used AS in some of the pairwise comparisons and applied statistical tests that are moderately robust to small sample sizes (see below). Similar considerations were also implemented in other microbiome studies of asthma and rhinitis including small groups or cohorts (Hilty et al., 2010; Castro-Nallar et al., 2015; Pérez-Losada et al., 2015; Lal et al., 2017; Fazlollahi et al., 2018; Pérez-Losada et al., 2023a,b).

### 2.3 High-throughput sequencing

Total DNA was extracted from swabs using the ZymoBIOMICS™ DNA Miniprep Kit D4300. DNA extractions were prepared for sequencing using the Schloss’ MiSeq_WetLab_SOP protocol in Kozich et al. (2013). DNA samples were amplified and sequenced for the ITS1-ITS2 region (~230 bp) following the protocols used in the Earth Microbiome Project (Thompson et al., 2017) and primer ITS1F Fwd: CTTGGTCATTTAGAGGAAGTAA and primer ITS2 Rev: GCTGCGTTCTTCATCGATGC—https://earthmicrobiome.org. All samples were sequenced in a single run of the Illumina MiSeq sequencing platform at the University of Michigan Medical School. Negative controls processed as above showed no PCR band on an agarose gel. We used eight water and reagent negative controls and five mock communities (i.e., reference samples with a known composition) to detect contaminating microbial DNA within reagents and measure the sequencing error rate. We did not find evidence of contamination and our sequencing error rate was as low as 0.0051%.

### 2.4 Mycobiome analyses

Internal transcriber spacer amplicon sequence variants (ASV) in each sample were inferred using dada2 version 1.18 (Callahan et al., 2016) and following author’s recommendations for the ITS region.^1^ Reads were filtered using standard parameters, with no uncalled bases, maximum of two expected errors and truncating reads at a quality score of 2 or less. Forward and reverse reads were merged and chimeras were identified. Taxonomic assignment was performed against the UNITE v9.0 2023-07-18 database (Nilsson et al., 2019) using the implementation of the RDP naive Bayesian classifier available in the dada2 R package (Wang et al., 2007; Quast et al., 2013). ASV sequences were aligned in MAFFT (Katoh and Standley, 2013) and used to build a tree with FastTree (Price et al., 2010). The resulting ASV tables and phylogenetic tree were imported into phyloseq (McMurdie and Holmes, 2013) for further analysis. Sequence files and associated metadata and BioSample attributes for all samples used in this study have been deposited in the NCBI (PRJNA1107919). Metadata and ASV abundances with corresponding taxonomic classifications are presented in Supplementary Tables S1, S2, respectively.

We normalized our samples using the negative binomial distribution (McMurdie and Holmes, 2014) implemented in the Bioconductor package DESeq2 (Love et al., 2014). This approach simultaneously accounts for library size differences and biological variability and has increased sensitivity if groups include less than 20 samples (Weiss et al., 2017). Taxonomic and phylogenetic alpha-diversity (within-sample) were estimated using Chao1 richness and Shannon, Abundance-based Coverage Estimator (ACE), and Phylogenetic Diversity (PD) indices. Beta-diversity (between-sample) was estimated using phylogenetic Unifrac (unweighted and weighted), Bray–Curtis and Jaccard distances, and dissimilarity between samples was explored using principal coordinates analysis (PCoA).

Differences in taxonomic composition (phyla and genera) and alpha-diversity indices between disease groups (AR, ARAS, and AS) and healthy individuals (CT) were assessed using linear models (mixed and standard) analysis to account for the non-independence of subjects (random effect)—lmer4 R package (Bates et al., 2015). We also included age, season and sex as covariables in all our initial model comparisons. Lineal models with randomized subjects were not better than those without random effects, as suggested by their similar or lower scores for the Akaike Information Criterion (AIC) and Bayesian Information Criterion (BIC). Additionally, none of the covariables were significant for any of the taxonomic and diversity indices compared. Beta-diversity indices were compared using permutational multivariate ANOVA (adonis)—vegan R package (Dixon, 2003). We applied the Benjamini–Hochberg method at alpha = 0.05 to correct for multiple hypotheses testing (Cook, 1977; Benjamini and Hochberg, 1995). All the analyses were performed in R (R Development Core Team, 2008) and RStudio (RStudio Team, 2015).

### 2.5 Functional analyses

Metabolic pathways were predicted by imputation of gene families and genomes as implemented in PICRUSt2 (Douglas et al., 2020). Briefly, we used the fungi ITS reference database provided by the developers to align our ITS sequences (minimum alignment 0.6) and then place them onto an ITS phylogenetic tree. Using ASV abundances obtained in dada2, we predicted gene family profiles and ultimately sample pathway abundances. Pathways were annotated using the MetaCyc database (Caspi et al., 2020) and differential pathway abundance among groups was determined in DESeq2 (Wald test; adjusted *p* value <0.01). Statistical analyses and visualization were conducted using functions in the ggpicrust R package (Yang et al., 2023).

### 2.6 Network analyses

Changes in fungal community structure were explored using covariation network analysis as implemented in Spiec-Easi (Kurtz et al., 2015). We estimated networks for AR, ARAS, and CT at the genus level (abundance filter threshold = 0.0005; mb method; greedy clustering). Network estimation, statistics, and visualization was carried out in the microeco R package (Liu et al., 2021).

## 3 Results

We collected nasal swabs from a cohort of 339 participants (214 individuals with respiratory disease and 125 healthy controls) from northern Portugal comprised mainly of children and young adults (Supplementary Table S1). The median age of the participants was 12.5 ± 5.0 years and 53.7% were female. Subjects with respiratory disease were subdivided into three groups: AR (47), ARAS (155), and AS (12 subjects). We sequenced the ITS1-ITS2 gene to characterize the nasal mycobiome of each participant. Twenty-two samples (i.e., technical replicates) from the following groups were sequenced twice due to seemingly faint PCR bands in agarose gels: AR (seven samples), ARAS (13 samples), and CT (two samples). ASV singletons and samples with <1,014 reads were eliminated, rendering a final data set of 306 samples with the following distribution: AR (42 samples from 36 individuals), ARAS (142 samples from 130 individuals), AS (12 samples from 12 individuals), and CT (110 samples from 108 individuals).

### 3.1 Mycobiome taxonomic diversity and structure

Our nasal mycobiome (306 samples after quality control) dataset comprised 6,145,342 clean reads, ranging from 1,014 to 223,989 sequences per sample (mean = 20,082.8) and a total of 5,635 ASVs (Supplementary Table S2). AR samples had 570 unique ASVs, ARAS samples had 2,202, AS samples had 138 and CT samples had 1,615 (Supplementary Figure 1). The four groups shared 122 ASVs, the disease groups shared 78 ASVs, while other pairs and trios shared a variable number, ranging from 1 to 323 ASVs (Supplementary Figure 1).

**Figure 1:**
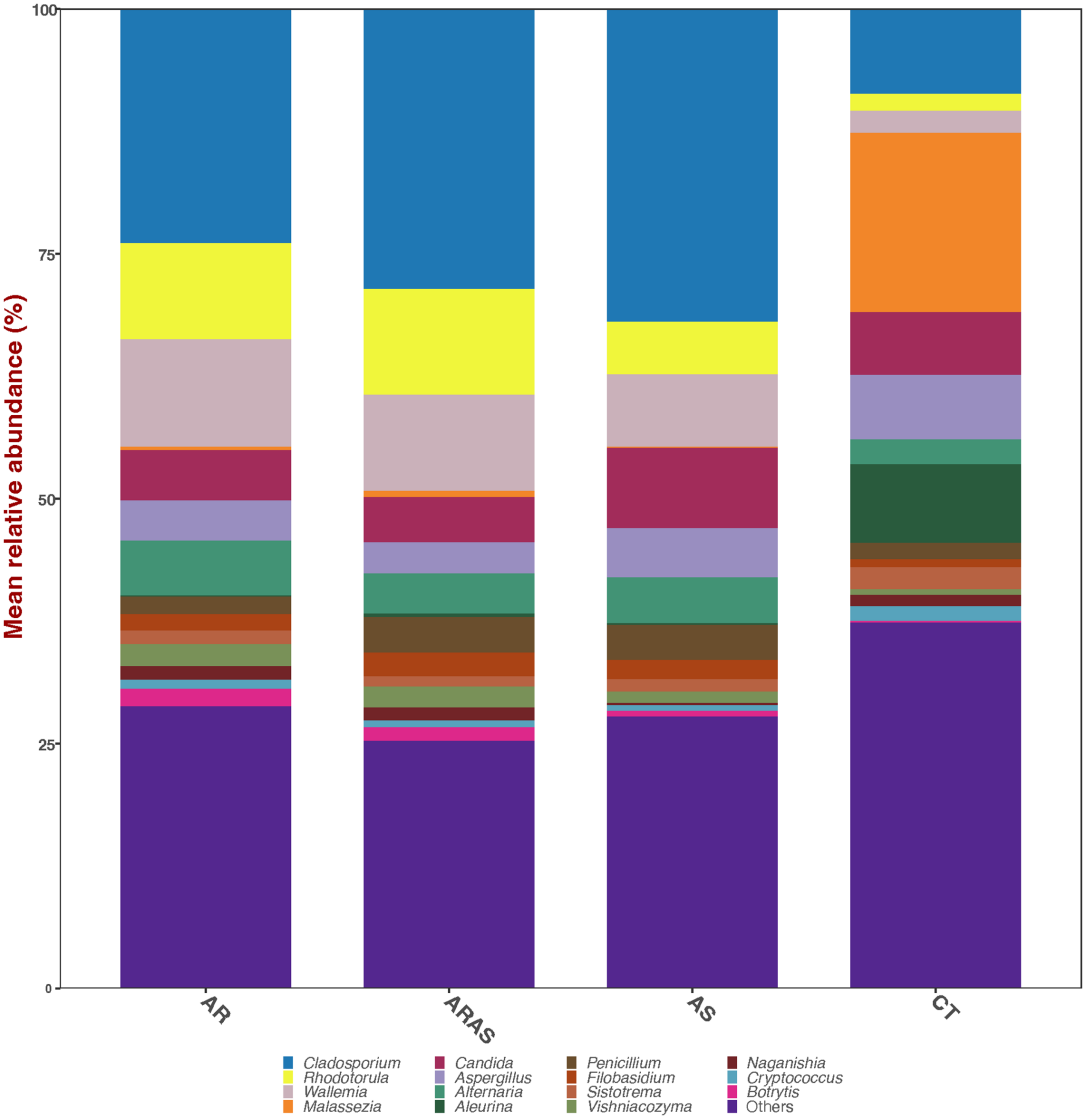
Alpha-diversity estimates (Chao1, Shannon, ACE, and phylogenetic diversity) and statistical significance (linear model test) in nasal fungal communities from participants with allergic rhinitis (AR), AR with comorbid asthma (ARAS), asthma (AS), and healthy controls (CT). ns, not significant; *p ≤ 0.05; ****p ≤ 0.0001.

The nasal mycobiome sequences across all 306 filtered samples were classified into two dominant (>1% abundance) Phyla: Ascomycota (54.0%) and Basidiomycota (44.9%) (Table 1). Those Phyla comprised 14 dominant (>1%) genera (Table 1; Figure 1), being the most abundant *Cladosporium* (23.0%), *Wallemia* (8.9%), *Malassezia* (8.3%), and *Rhodotorula* (8.2%). All the other detected phyla and genera accounted for <1% of the total ITS sequences each.

**Table 1:** Mean relative proportions (%) of fungal phyla and genera in the nasal mycobiome of participants with allergic rhinitis (AR), AR with comorbid asthma (ARAS), asthma (AS) and healthy controls (CT).

		Mean relative proportions (%)						Linear model test significance			
	All	AR	ARAS	AS	CT	AR-CT	ARAS-CT	AS-CT	AR-ARAS	AR-AS	ARAS-AS
Phylum											
Ascomycota	54	49.2	56.2	79.2	48.6	ns	ns	ns	ns	ns	ns
Basidiomycota	44.9	49.6	43.3	20.4	49.4	ns	ns	ns	ns	ns	ns
Genus											
*Malassezia*	8.3	0.3	1.3	0.2	23.7	<0.0001	<0.0001	<0.0001	ns	ns	ns
*Alternaria*	3.2	3.6	3.6	1.6	2.7	0.024	0.0003	ns	ns	ns	ns
*Cladosporium*	23	23.3	28.6	63.6	7.5	<0.0001	<0.0001	<0.0001	ns	ns	ns
*Penicillium*	2.4	1.8	3.3	1.4	1.6	0.049	<0.0001	0.031	ns	ns	ns
*Aspergillus*	4	3.6	3.4	1.9	5.4	ns	ns	ns	ns	ns	ns
*Candida*	4.7	4.2	4.7	4.6	5.2	ns	ns	ns	ns	ns	ns
*Aleurina*	2.4	0.1	0.2	0.3	7	ns	ns	ns	ns	ns	ns
*Wallemia*	8.9	15.8	11.8	2.7	2.5	<0.0001	<0.0001	0.0063	ns	ns	ns
*Rhodotorula*	8.2	12.2	12.1	3.5	1.6	<0.0001	<0.0001	0.0008	ns	ns	ns
*Sporobolomyces*	1.2	1.1	2	0.9	0.1	<0.0001	<0.0001	ns	ns	ns	ns
*Naganishia*	1.4	2.9	1.2	0.4	1	0.016	0.0026	ns	ns	ns	ns
*Vishniacozyma*	1.4	2.9	1.5	0.9	0.6	<0.0001	<0.0001	0.0023	ns	ns	ns
*Sistotrema*	1	0.5	0.6	1.5	1.7	ns	ns	ns	ns	ns	ns
*Filobasidium*	1.4	1.5	1.4	2.8	1.1	0.0009	<0.0001	0.019	ns	ns	ns

*p* values for significant pairwise comparisons (linear model test) between groups are also displayed. ns, not significant.

ASV2 of the genus *Cladosporium* comprised the nasal core microbiome (prevalence ≥90%) of the respiratory disease patients and accounted for 12.8% of their total reads. No core mycobiome was detected for the control samples. ASV2 may represent the more stable and consistent member of the nasal mycobiomes (Backhed et al., 2012; Shade and Handelsman, 2012) in the disease patients.

We also compared the mean relative abundance of specific taxa in subjects with respiratory disease and healthy controls. None of the two dominant fungal phyla (Ascomycota and Basidiomycota) comprising the nasal microbiome showed significant differences in their mean relative proportions between the groups compared (Table 1). Of the 14 dominant fungal genera comprising the nasal microbiome (Figure 1; Table 1), 7–10 genera showed significant differences in their mean relative proportions between all respiratory disease group (AS, AR or ARAS) and CT after FDR correction. However, none of the same genera varied significantly between the three respiratory disease groups (Table 1).

**Figure 2:**
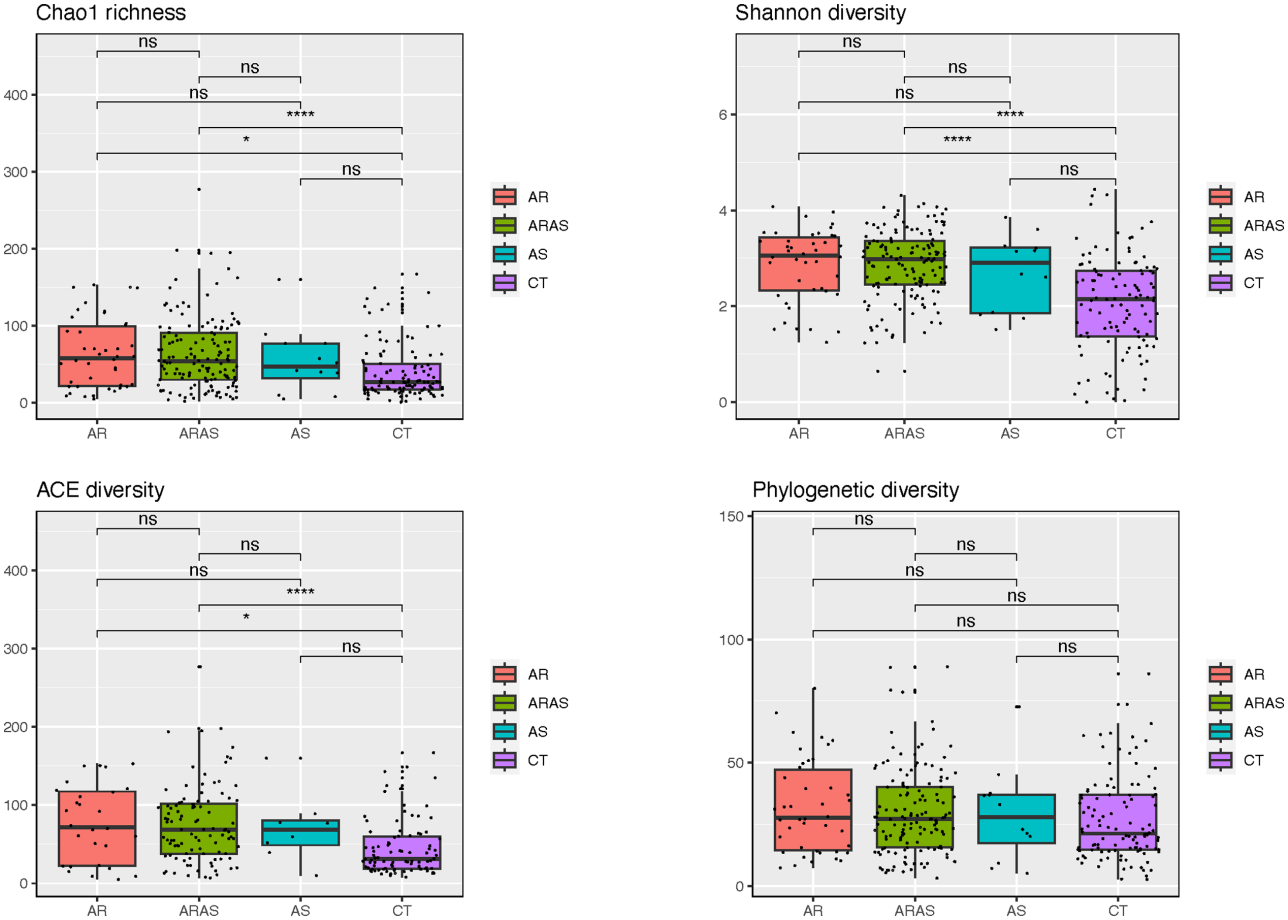
Bar plots of mean relative proportions of the top fungal genera in the nasal cavity of participants with allergic rhinitis (AR), AR with comorbid asthma (ARAS), asthma (AS), and healthy controls (CT).

Alpha-diversity indices (Shannon, Chao1, ACE, and PD) of microbial community richness and evenness varied among clinical groups (Figure 2; Supplementary Table S3). AR and ARAS showed the highest diversity for all indices, while CT showed the lowest. ARAS–CT and AR–CT comparisons were significantly distinct for Shannon, Chao1, and ACE after FDR correction (Wilcoxon test; *p* ≤ 0.024). All the other pairwise comparisons, including those of PD estimates, were not significant.

**Figure 3:**
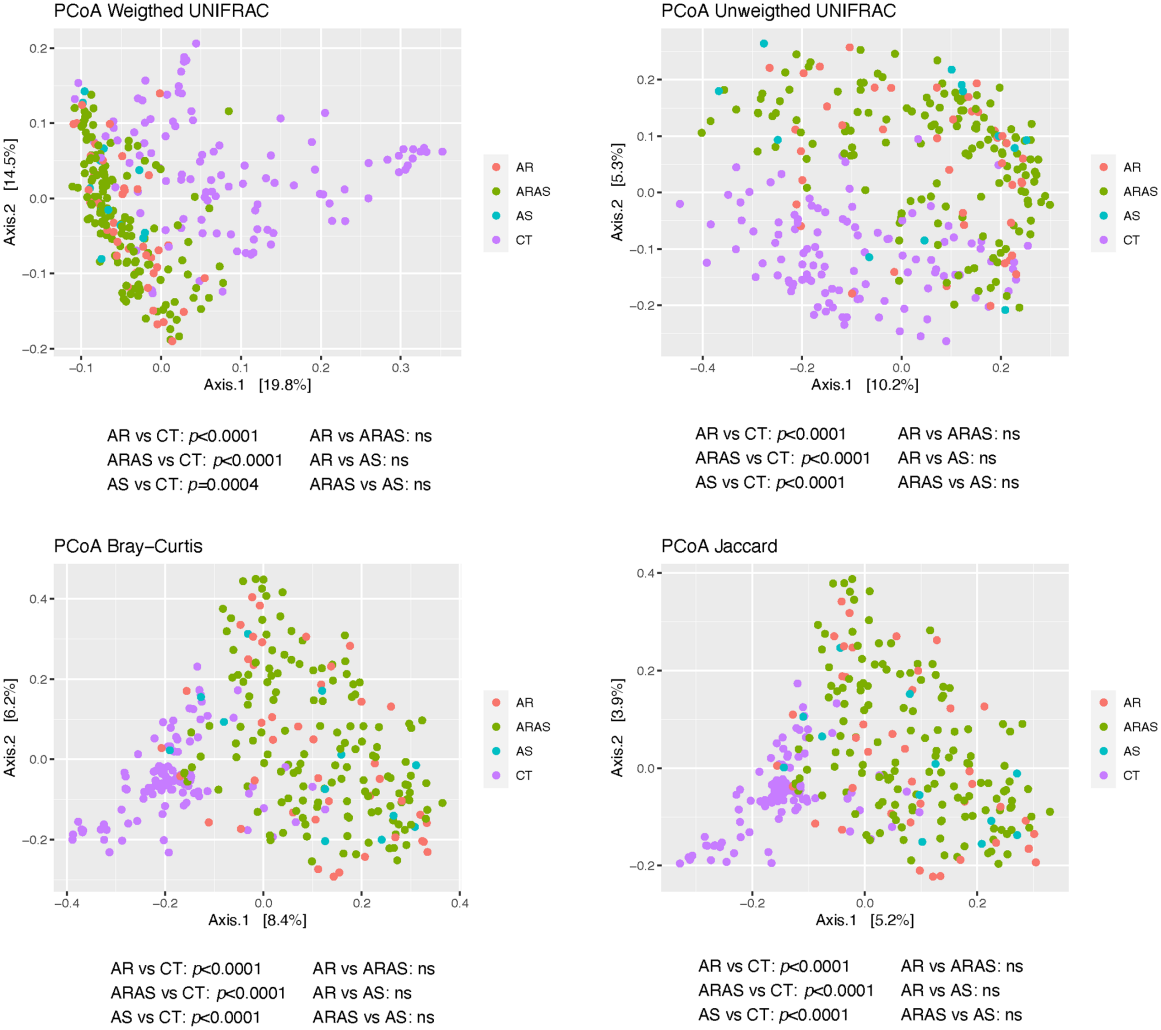
Principal coordinates analysis (PCoA) plots of beta-diversity estimates (Unifrac, Bray-Curtis and Jaccard indices) and statistical significance (Adonis test) in nasal fungal communities from participants with allergic rhinitis (AR), AR with comorbid asthma (ARAS), asthma (AS), and healthy controls (CT). ns, not significant.

To characterize the structure of the nasal mycobiomes (beta diversity), we applied principal coordinates analysis (PCoAs) to Unifrac (unweighted and weighted), Bray–Curtis and Jaccard distance matrices. All the PCoAs showed partial segregation of the mycobiotas from each clinical group (Figure 3). Subsequently, adonis analyses detected significant differences (*p* ≤ 0.0004) in beta-diversity between each of the respiratory disease groups (AS, AR and ARAS) and the healthy controls for all the distances. None of the pairwise comparisons between respiratory disease groups resulted significant. This suggests that the nasal mycobiomes of AS, AR and ARAS participants may differ from those of healthy individuals in a similar compositional manner.

### 3.2 Mycobiome functional diversity

**Figure 4:**
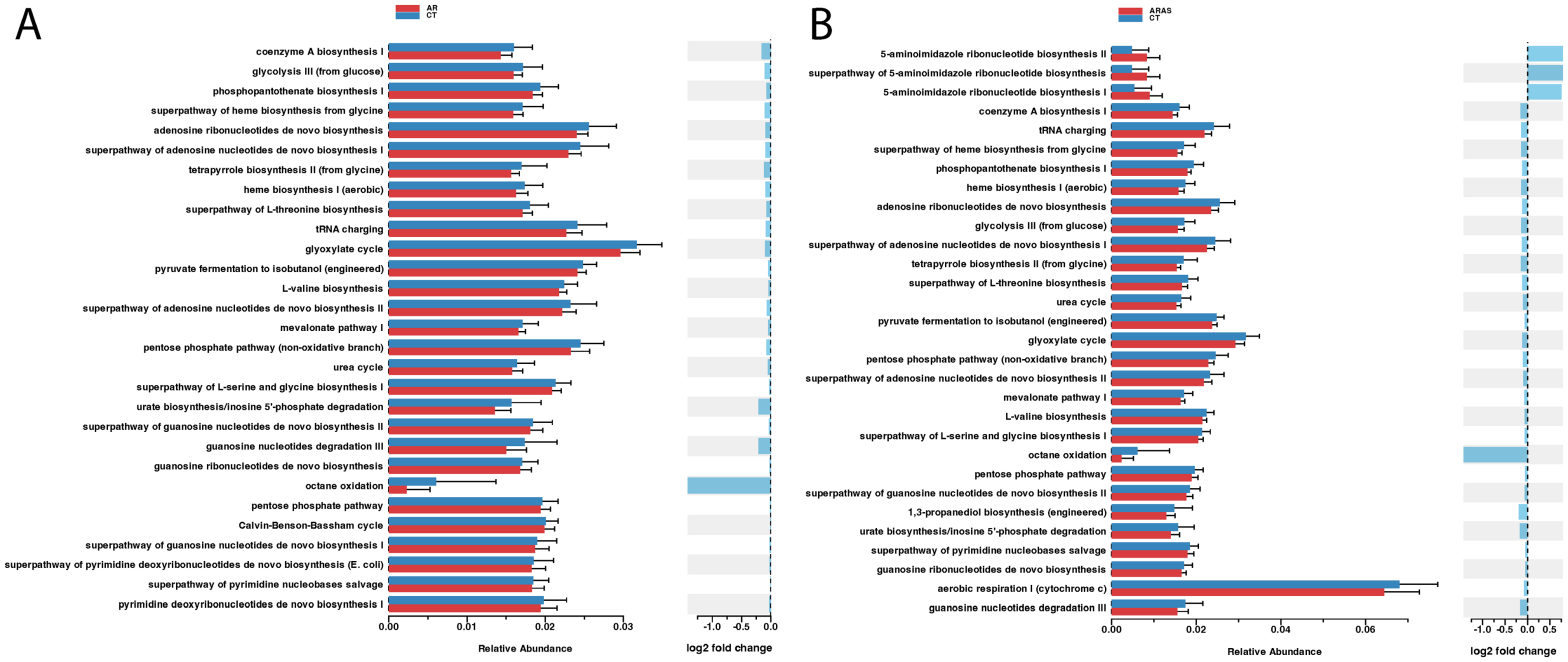
Spiec-Easi networks of fungal taxa in the nasal mycobiomes of participants with allergic rhinitis (AR), AR with comorbid asthma (ARAS) and healthy controls (CT). Nodes represent taxa connected by edges whose width (0.1–0.4) is proportional to the strength of their association. Cyan and pink edges indicate positive and negative correlations, respectively.

To understand whether different disease groups exhibited differences in the nasal mycobiome functional capabilities, we inferred the functional potential of AR, ARAS, and CT groups. We found significant differences (adjusted *p*-value <0.01) in abundance in 30 pathways (MetaCyc annotated) between AR and CT or ARAS (Figure 4). Most changes in pathway abundance represented pathways enriched in CT compared to AR or ARAS with negative or nearly zero log2 Fold Change (FC). Only three pathways associated with 5-aminoimidazole ribonucleotide biosynthesis were over abundant (log2 FC > 0.75) in ARAS patients (Figure 4B). These pathways are associated with the *de novo* biosynthesis of purine nucleotides and of thiamin (PWY-6121; PWY-6122; PWY-6277). Interestingly, the comparison AR versus ARAS yielded no significant results (*p*-value >0.1), suggesting both conditions share a similar nasal mycobiome functional signature.

### 3.3 Mycobiome interactions

**Figure 5:**
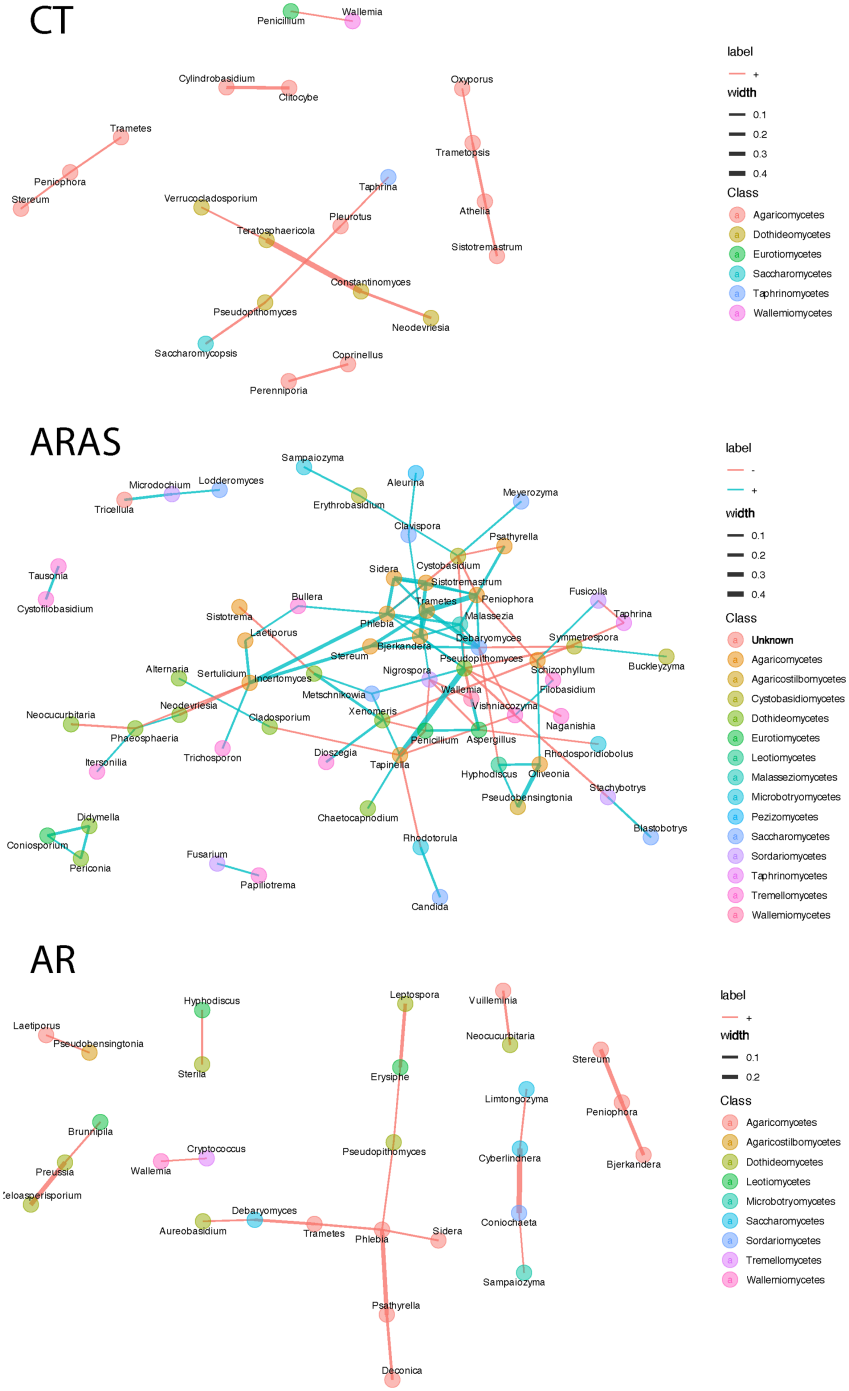
Differential abundance analysis (Wald’s test; adjusted *p* value <0.01) of functional profiles in the nasal mycobiomes of participants with allergic rhinitis (AR) and healthy controls (CT) **(A)**, and AR participants with comorbid asthma (ARAS) and CT **(B)**.

We further wanted to investigate potential direct or indirect interactions among fungal groups. We inferred inverse covariance networks using the Spiec-Easi model to compare the structure and connectivity of the nasal mycobiome. In the CT network, we identified seven modules of interacting fungi (Figure 5) with a degree of connectivity between 1 and 2, indicating very low connectivity. Likewise, betweenness centrality, a measure of importance of a node in a network, was also low (range 0–2). In turn, in the ARAS network (Figure 5), degree of connectivity ranged between 1 and 7, indicating higher connectivity. Some fungal genera were connected up to other 7 genera, and of those, *Cystobasidium*, *Pseudopithomyces*, *Peniophora*, and *Debaryomyces*, presented high betweenness centrality (e.g., *Peniophora* > 90), highlighting their role as hubs in the ARAS mycobiome. The AR network showed a degree of connectivity and betweenness centrality of 1–4 and 0–30, respectively (Figure 5). It shared similarities with the ARAS network, where *Phlebia* and *Debaryomyces* were also highly connected (4 and 5 in AR; 2 and 5 in ARAS). Node overlap between the three networks varied; ARAS and AR shared 23.6% of the nodes, ARAS and CT shared 14.6% and AR and CT shared 9.1%. Edge overlap was limited between networks (<5%), suggesting the overall structure of the networks is different.

## 4 Discussion

The role of the fungal communities residing in the upper airways in allergic rhinitis and asthma is practically unknown (Jung et al., 2015; Yuan et al., 2023). Here we present the results of a cross-sectional study comparing the nasal mycobiome of 339 individuals with allergic rhinitis (with and without comorbid asthma), asthma and healthy controls.

The nasal mycobiomes were composed of basically two phyla (Ascomycota and Basidiomycota) and 14 genera (Figure 1; Table 1). These two phyla and all dominant genera have been previously described in the airways of both healthy, asthmatic and rhinitic individuals, although with different abundances (Jung et al., 2015; Carpagnano et al., 2016; Goldman et al., 2018; Rick et al., 2020; van Tilburg Bernardes et al., 2020; Yuan et al., 2023). We detected common opportunistic pathogenic fungi like *Malassezia, Aspergillus, Candida*, and *Penicillium* (Badiee and Hashemizadeh, 2014). Moreover, exposure to *Alternaria* spores has been associated with AR symptoms (Andersson et al., 2003; Oliveira et al., 2023). This confirms at fungal level what is already known for bacteria, that the nasal cavity is a major reservoir for opportunistic pathogens that can cause allergic rhinitis and asthma (Garcia-Rodriguez and Fresnadillo Martinez, 2002; Hilty et al., 2010; Bogaert et al., 2011; Dickson et al., 2013; Biesbroek et al., 2014; Huang and Boushey, 2015; Pérez-Losada et al., 2015; Teo et al., 2015; Pérez-Losada et al., 2016b; Prevaes et al., 2016; Huang, 2017; Lal et al., 2017; Pérez-Losada et al., 2017; Esposito and Principi, 2018; Pérez-Losada et al., 2018; Gan et al., 2021; Chen et al., 2022; Kim et al., 2022; Pérez-Losada et al., 2023a).

Healthy participants differed greatly in fungal composition from those with chronic respiratory illnesses. The nasal mycobiome of healthy controls contained 28.7% unique ASVs, while the AR, ARAS and AS mycobiomes contained 10.1, 39.1, and 2.4% unique ASVs, respectively (Supplementary Table S2; Supplementary Figure 1). These ASVs are potential biomarkers of disease for each group. Further metataxonomic and metagenomic studies are needed to confirm these results and their potential as therapeutic targets for rhinitis and asthma (Castro-Nallar et al., 2015; Pérez-Losada et al., 2015; Pérez-Losada et al., 2023a,b).

Fungal phyla (Ascomycota and Basidiomycota) did not vary significantly in their mean relative proportions between groups, but up to 71% of the dominant genera varied significantly between healthy samples and respiratory disease groups (Table 1). The most striking differences were observed between AR or ARAS and CT, where 10 of 14 genera varied in their mean relative abundances, respectively. *Alternaria, Cladosporium, Penicillium, Wallemia, Rhodotorula, Sporobolomyces, Naganishia, Vishniacozyma* and *Filobasidium* were significantly more abundant in AR and ARAS, while *Malassezia* was significantly more abundant in healthy controls. A previous study of the nasal vestibule (Jung et al., 2015) in four patients with allergic rhinitis and four controls showed that Basidiomycota and *Malassezia* were highly abundant in all samples (>92%); nonetheless, no significant differences were reported among groups. This disagrees with our findings here and could result from the low sample size, fungal gene sequenced (i.e., large ribosomal subunit) or geographic region (i.e., Seoul metropolitan area) in Jung et al.’s study. Seven fungal genera varied significantly between AS and CT, despite the small sample size of this group. As before, *Malassezia* was also much more abundant in CT, while the other genera varied less in their mean relative abundances. Previous studies have also revealed significant differences in the mycobiota of asthmatic patients for *Cladosporium, Rhodotorula, Malassezia* or *Penicillium* (van Woerden et al., 2013; Goldman et al., 2018; Sharma et al., 2019; Yuan et al., 2023). Compositional changes in these fungal groups may provide insights into the pathobiology of allergic rhinitis and asthma. Further studies are required to confirm our findings and untangle the relationship between fungal colonization, dysbiosis and chronic inflammatory disease (Nguyen et al., 2015; Goldman et al., 2018; Rick et al., 2020; van Tilburg Bernardes et al., 2020; Yuan et al., 2023).

Fungal alpha-diversity (species richness and evenness) was significantly higher in ARAS and AR compared to CT for all indices but PD (Figure 2). The only study that explored the diversity of the nasal mycobiota in individuals with rhinitis (Jung et al., 2015) also reported higher estimates of Shannon diversity for the AR group. If confirmed, this may suggest that allergic rhinitis (with or without asthma comorbidity) may increase microbial diversity in the upper airways, as seen in previous studies of the bacteriome (Choi et al., 2014; Gan et al., 2021; Kim et al., 2022; Pérez-Losada et al., 2023a,b).

AR, ARAS, and AS samples displayed significant differences in community structure (i.e., beta-diversity) compared to those of healthy controls (Figure 3). This pattern held for all the distance metrics used, whether accounting for phylogenetic diversity or not. No differences were observed between AR and ARAS groups. A previous study of the nasal mycobiota (Jung et al., 2015) has also revealed that AR and CT communities were considerably differentiated. Another study (2020) has also shown specific community structuring associated with distinct bacterial composition of the lung in AS vs. CT. Hence, as indicated before (Pérez-Losada et al., 2023a,b), these results suggest that fungal compositional shifts may be a reliable predictor of allergic rhinitis or asthma in the upper airways, given their lower stochasticity associated to dysbiosis (Ma et al., 2019; Ma, 2020).

The functional component of the allergic rhinitis mycobiome is largely underexplored. Here, we used an imputation method to indirectly explore the functional potential of the nasal mycobiome (Figure 4). We found modest yet significant differences in metabolic pathway abundance when comparing the AR to CT groups. Pathway relative overexpression was high for three pathways related to 5-aminoimidazole ribonucleotide (AIR) biosynthesis in the ARAS group (log2 FC > 0.75). AIR is a key intermediate for purine nucleotide biosynthesis and a precursor to 4-amino-2-methyl-5-hydroxymethylpyrimidine, the first product of pyrimidine biosynthesis. No studies so far have investigated AIR biosynthesis in the airway microbiome, but, interestingly, studies of the gut bacteriome have related AIR biosynthesis to several clinical conditions and diseases (hyperuricemia, inflammatory bowel disease and colorectal cancer) (Ma et al., 2021; Sheng et al., 2021). The impact (if any) of fungal AIR biosynthesis in human health has not been investigated. Purine metabolism is necessary to synthesize DNA and RNA, and in plant pathogenic fungi is associated with fungal growth and pathogenesis (Sun et al., 2024). Some authors (Chitty and Fraser, 2017) have reviewed the literature regarding purine acquisition and synthesis in human pathogenic fungi, finding that purines are essential in diverse processes such as signal transduction, energy metabolism and DNA synthesis, turning AIR biosynthesis into a potential therapeutic target. More studies are needed to test whether AIR biosynthesis in the human airway mycobiome is associated with respiratory diseases such as allergic rhinitis or asthma.

We have also explored mycobiome interactions to better understand the role of fungi in the nasal cavity (Figure 5). Direct or indirect interactions are usually inferred based on co-occurrence or co-variation of microbes’ abundance. For instance, positive interactions might be indicative of syntrophy (a relationship in which one or both organisms benefit nutritionally from the presence of the other), while negative interactions may indicate competition. The CT and AR groups showed fewer significant interactions, all of which were positive, suggesting either similar roles of fungi in the community or syntrophy. In turn, the ARAS group exhibited more diverse relationships with multiple modules with positive and negative interactions among fungal taxa. Previous research has shown that these patterns of co-abundance and exclusion seem to be stable across body sites in the healthy human microbiome and that its alteration can be indicative of underlying disease processes (Faust et al., 2012). In previous studies of the bacteriome in patients with allergic rhinitis (Pérez-Losada et al., 2018; Pérez-Losada et al., 2023a,b) or of the mycobiome in asthmatics (Huang et al., 2020; Liu et al., 2020), co-occurrence networks in diseased participants exhibited different interactions than in healthy controls. Our novel analyses of the airway mycobiome in rhinitic patients seem to confirm those results, although with the allergic rhinitis and comorbid asthma group (ARAS) exhibiting a higher and more diverse mycobiome network. Interestingly, in spite of the multiple connections of rhinitis and asthma and the proposed concept of a united airway disease (Compalati et al., 2010), recent omic data (Dizier et al., 2007; Lemonnier et al., 2020) suggest that rhinitis alone and rhinitis with comorbid asthma may represent two distinct diseases with different allergen sensitization and disease onset (Siroux et al., 2018), rhinitis severity (Savoure et al., 2023) and treatment response (Sousa-Pinto et al., 2022). Moreover, the hypothesis that these two distinct diseases are possibly modulated by the microbiome has been recently proposed (Bousquet et al., 2023). Further research is needed to explore the role of fungi in chronic inflammation, particularly in allergic individuals.

Our study highlights significant differences in the nasal mycobiome composition, structure, and function between individuals with allergic rhinitis and asthma and healthy controls. These findings have profound implications for understanding innate and adaptive host immune responses to fungi in the airways (Bartemes and Kita, 2018; Silva-Gomes et al., 2024). The nasal mycobiome can modulate the local immune environment. Fungal components, such as cell wall polysaccharides (e.g., β-glucans), are known to interact with pattern recognition receptors on immune cells, leading to the activation of various immune pathways (Brown et al., 2012; Bartemes and Kita, 2018). This interaction can exacerbate or alleviate inflammation in the respiratory tract, influencing the severity of allergic reactions. Altered nasal mycobiome profiles, such as those revealed here, may contribute to allergic sensitization. Fungi can also produce potent allergens that trigger Th2-mediated immune responses, characterized by increased production of IgE and activation of mast cells and eosinophils (Noverr and Huffnagle, 2004; Noverr et al., 2004; Bartemes and Kita, 2018). This immune activation plays a critical role in the pathogenesis of allergic rhinitis and asthma. The nasal epithelial barrier’s integrity and the effectiveness of innate immune defenses are closely linked to mycobiome composition. Dysmycobiosis, i.e., imbalance in the fungal community, can compromise these defenses, making individuals more susceptible to infections and exacerbations of allergic conditions (Iliev and Leonardi, 2017). Our findings are supported by previous research demonstrating that distinct nasal mycobiome profiles can activate different immunological responses. For instance, van Woerden et al. (2013) showed that fungal dysbiosis in asthmatic patients correlates with altered immune responses, including increased airway inflammation. A recent review (Jafarlou, 2024) has highlighted that fungal diseases are emerging as a significant global health threat, with the potential to cause a pandemic with widespread outbreaks and significant morbidity and mortality. There is already growing evidence that the lung mycobiome has a significant impact on clinical outcome of chronic respiratory diseases such as asthma (Nguyen et al., 2015). Little is known, however, about allergic rhinitis or the role of the nasal cavity mycobiota (Jung et al., 2015). We showed that nasal dysmycobiosis may contribute to allergic rhinitis with or without asthma comorbidity and warrants further research to elucidate the relationship between the nasal mycobiota and airway pathology.

This study has several limitations. Metataxonomic approaches suffer from the inherent limitations of collecting sequence data from a single gene target (ITS here) (Hilton et al., 2016; Pérez-Losada et al., 2022). PCR amplification biases can also impact microbial compositional assessments. ITS1-2 has limited resolution at the species and sometimes genus level for taxonomic assignment. Although the composition of the described nasal mycobiomes is similar to those reported by others in the nasal cavity of healthy and diseased individuals (Jung et al., 2015; Carpagnano et al., 2016; Goldman et al., 2018; Rick et al., 2020; van Tilburg Bernardes et al., 2020; Yuan et al., 2023). The sample size of the asthmatic (AS) group is relatively small, although we have tried to account for it using statistical approaches moderately robust to small sample sizes. The metabolic potential of the mycobiomes was predicted by imputation of gene families and genomes in PICRUSt2 instead of inferred using shotgun metagenomics; hence functional profiles should be interpreted with caution. This study focuses on a cohort of Portuguese individuals for whom we have collected limited demographic and clinical data (i.e., heath status, season, age, and sex) for all the participants. It is uncertain to what extent our results can be generalized to other countries and cohorts, but since clinical practices in Portugal for treating rhinitis and asthma follow international guidelines and recommendations and nasal mycobiomes characterized here resembled those described in other studies of cohorts from United States, Europe, and Asia, we feel like our insights are broadly applicable. Nonetheless, future research should address the impact of other demographic, clinical and environmental factors on the diversity of airway mycobiomes (Cavaleiro Rufo et al., 2017; Zhang et al., 2023; Paciencia et al., 2024). The relevance of detecting fungi associated with specific phenotypes of disease is unknown, dual-transcriptomic studies coupled with longitudinal sampling (as opposed to the cross-sectional sampling design used here) can help to clarify whether specific microbes are drivers or bystanders in rhinitic and asthmatic patients. Future microbiome research should address this issue.

## Data Availability

The datasets presented in this study can be found in online repositories. The names of the repository/repositories and accession number(s) can be found at: https://www.ncbi.nlm.nih.gov/, PRJNA1107919.

## Footnotes

^1^ https://benjjneb.github.io/dada2/ITS_workflow.html

## Supplementary material

SUPPLEMENTARY FIGURE 1 UpSet plots of amplicon sequence variants (ASVs) in the nasal mycobiome of participants with allergic rhinitis (AR), AR with comorbid asthma (ARAS), asthma (AS) and healthy controls (CT).
