# Supplementary figures and images for: The nasal mycobiome of individuals with allergic rhinitis and asthma differs from that of healthy controls in composition, structure and function
Source: Front Microbiol. 2024 Dec 17;15:1464257. doi: 10.3389/fmicb.2024.1464257 (PMC11685215; doi:10.3389/fmicb.2024.1464257)

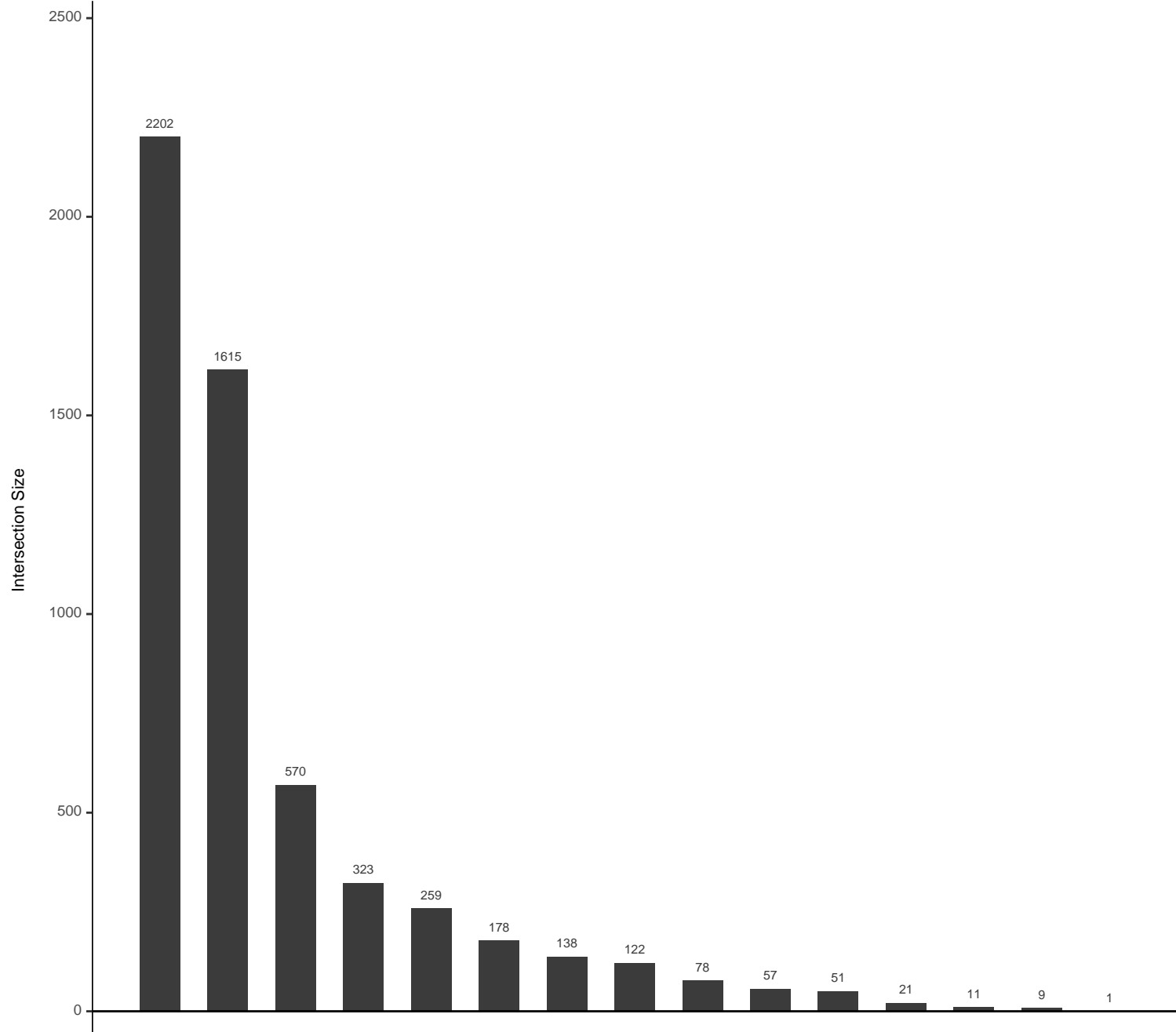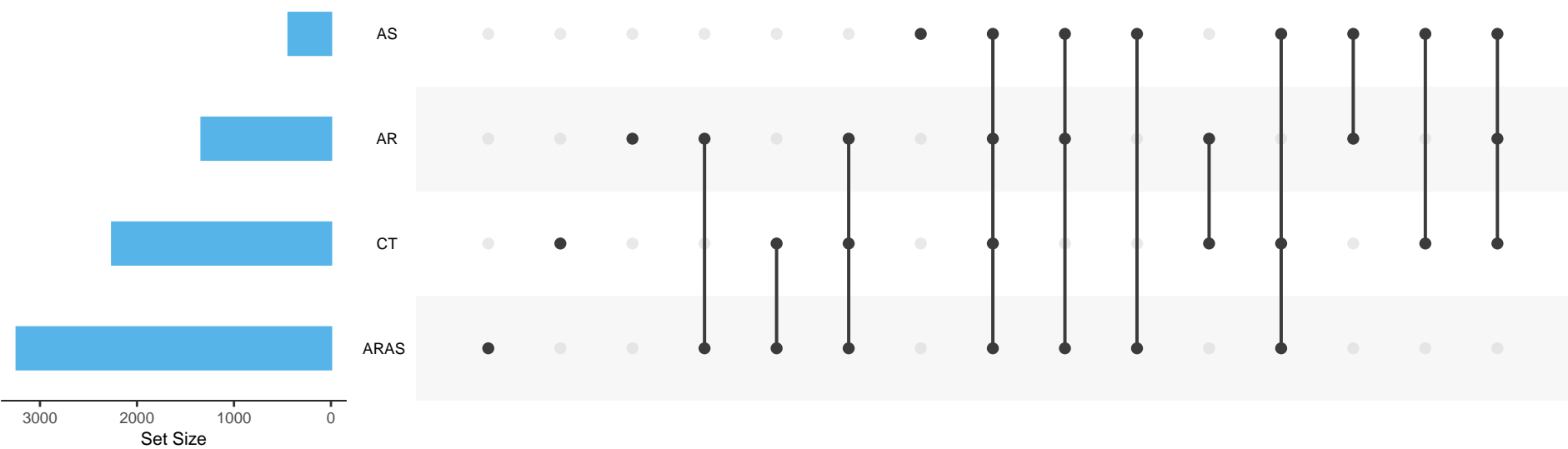

Supplement: Supplementary file 1 [file Data_Sheet_1.pdf]
